# USA National Phenology Network’s volunteer-contributed observations yield predictive models of phenological transitions

**DOI:** 10.1371/journal.pone.0182919

**Published:** 2017-08-22

**Authors:** Theresa M. Crimmins, Michael A. Crimmins, Katharine L. Gerst, Alyssa H. Rosemartin, Jake F. Weltzin

**Affiliations:** 1 National Coordinating Office, USA National Phenology Network, Tucson, Arizona, United States of America; 2 School of Natural Resources and the Environment, University of Arizona, Tucson, Arizona, United States of America; 3 Department of Soil, Water and Environmental Science, University of Arizona, Tucson, Arizona, United States of America; 4 U.S. Geological Survey, Tucson, Arizona, United States of America; Universite du Quebec a Chicoutimi, CANADA

## Abstract

**Purpose:**

In support of science and society, the USA National Phenology Network (USA-NPN) maintains a rapidly growing, continental-scale, species-rich dataset of plant and animal phenology observations that with over 10 million records is the largest such database in the United States. The aim of this study was to explore the potential that exists in the broad and rich volunteer-collected dataset maintained by the USA-NPN for constructing models predicting the timing of phenological transition across species’ ranges within the continental United States. Contributed voluntarily by professional and citizen scientists, these opportunistically collected observations are characterized by spatial clustering, inconsistent spatial and temporal sampling, and short temporal depth (2009-present). Whether data exhibiting such limitations can be used to develop predictive models appropriate for use across large geographic regions has not yet been explored.

**Methods:**

We constructed predictive models for phenophases that are the most abundant in the database and also relevant to management applications for all species with available data, regardless of plant growth habit, location, geographic extent, or temporal depth of the observations. We implemented a very basic model formulation—thermal time models with a fixed start date.

**Results:**

Sufficient data were available to construct 107 individual species × phenophase models. Remarkably, given the limited temporal depth of this dataset and the simple modeling approach used, fifteen of these models (14%) met our criteria for model fit and error. The majority of these models represented the “breaking leaf buds” and “leaves” phenophases and represented shrub or tree growth forms. Accumulated growing degree day (GDD) thresholds that emerged ranged from 454 GDDs (*Amelanchier canadensis*-breaking leaf buds) to 1,300 GDDs (*Prunus serotina*-open flowers). Such candidate thermal time thresholds can be used to produce real-time and short-term forecast maps of the timing of these phenophase transition. In addition, many of the candidate models that emerged were suitable for use across the majority of the species’ geographic ranges. Real-time and forecast maps of phenophase transitions could support a wide range of natural resource management applications, including invasive plant management, issuing asthma and allergy alerts, and anticipating frost damage for crops in vulnerable states.

**Implications:**

Our finding that several viable thermal time threshold models that work across the majority of the species ranges could be constructed from the USA-NPN database provides clear evidence that great potential exists this dataset to develop more enhanced predictive models for additional species and phenophases. Further, the candidate models that emerged have immediate utility for supporting a wide range of management applications.

## Introduction

Phenology, the timing of life cycle events in plants and animals, is responsive in many species to immediate environmental conditions such as temperature and precipitation [1, 2, 3]. Predicting when a species will undergo a phenological transition at a particular location—for example, transitioning from closed flower buds to open flowers—has great value for a wide range of short-term natural resource management applications. Optimal timing of management activities such as pest, pathogen, and invasive species detection or treatment; thinning, burning, and chemical treatments; and planting and harvest activities can all benefit from real-time information and short-term forecasts of phenological transitions [4, 5]. Further, advance knowledge of the timing of events in key species such as flowering in ornamental trees or leaf color change in deciduous trees can improve planning for recreational activities [6].

The USA National Phenology Network (USA-NPN) was established in 2007 to meet the growing needs for phenological data and derived products within the United States [7]. The USA-NPN collects, stores, and shares observations of plant and animal phenology to support and enhance management decisions, and to increase awareness of phenology, its relationship to environmental conditions and its influence on ecosystems [8]. In particular, the USA-NPN aims to produce national- to continental-scale data and information including near real-time maps and short-term predictions of the timing of phenological transitions to support a broad ranges of uses in sectors encompassing natural resources, human health, and agriculture.

As of July, 2017, the USA-NPN housed over 10 million records of plant and animal phenology from across the U.S. in the National Phenology Database (NPDb); these data can serve as the raw materials for establishing environmental drivers to phenological transitions in individual species. Once the relationships between environmental conditions and the timing of phenological transitions in individual species are established, these models can be used to produce real-time and short-term forecast maps of the timing of such transitions to directly support natural resource decision-making. Several recent studies have used data maintained by the USA-NPN to identify triggers to phenological transitions [9–14], though these studies have focused on a small number of species and phenophases.

For many species, the cues to phenological transition are unknown. Determining cues and thereby establishing predictive models of phenophase transitions requires ample reports of the date of phenological transitions for a particular species with high temporal precision. Often, such models are constructed using long-term records from a single location or region [1, 2, 15, 16], limiting their applicability for other locations. Alternatively, records with short temporal depth representing multiple locations can be used [13, 17, 18], though pooling observations from different portions of a species range can introduce further complexities, because differences in phenology observed across the range may reflect local adaption [19]. The ideal dataset for determining cues to phenological transitions for a species would be comprised of repeated observations of individual plants over a long period of time, with high temporal frequency, at many locations distributed across the species range—ultimately, what the USA-NPN endeavors to assemble.

The goal of this study was to explore the potential of the data maintained in the NDPb for establishing models of phenophase transition to be used to generate real-time and predictive maps [20]. The data housed in the NPDb are contributed by thousands of volunteers and professional natural resource managers and scientists [21]. As such, this data resource is species-rich and represents nearly 10,000 observation locations from across the United States [22]. However, these data have been collected opportunistically and are geographically unbalanced: nearly half of all USA-NPN sites reporting phenology data are located within an urban area (unpubl data). Further, the extent to which individual plants are observed in consecutive years is low, and observations may be collected with inconsistent frequency within a year. The precision around the date that a transition is captured is within 7 days for about half of estimated onset dates in the NPDb [23]. It is not known the extent to which these characteristics impact the suitability of the dataset for constructing predictive models.

We constructed simple thermal time models of phenological transitions using data housed in the NPDb for as many species and spring- and summer-season phenophases as possible. We implemented a single formulation of thermal time models because our focus was on exploring the potential for constructing models and generating predictive maps using the data, rather than definitively determining the cues to phenological transitions in various species. Thermal time is a viable option for this study because temperature performs at least as well as more complex model formulations at predicting the timing of phenological transitions in many temperate plant species’ spring- and summer-season phenophases [3, 13, 16, 24, 25].

Our objectives for this study were 1) to construct simple thermal time models of phenological transitions using data housed in the NPDb for as many species and spring- and summer-season phenophases was as possible given available data, and 2) to identify “candidate” thermal time models that could be used to produce predictive maps of phenological transition. The USA-NPN produces daily real-time and short-term forecast maps of accumulated growing degree days (AGDD; [20]) and the Extended Spring Indices [26–28] for the United States; the workflow employed to generate these maps could be readily extended to phenological transitions that are cued by accumulated heat. We also expected this effort to reveal species × phenophase combinations for which increased sampling frequency, additional sampling locations, or additional driving variables may be necessary to yield viable predictive models. Further, we anticipated that this analysis might reveal species in which local adaptation is at play, necessitating an alternative approach for predictive modeling.

## Materials and methods

### Phenology data

The phenology data maintained by the USA-NPN are contributed by thousands of volunteer citizen scientists from across the United States through the phenology observing program, *Nature’s Notebook* [21]. Over 1,200 plant and animal species are available for monitoring using vetted standardized phenophase definitions and protocols [29].

Data housed within the USA-NPN’s National Phenology Database are structured as “status” data, meaning that on each date an observation of an individual plant is made, the status of a phenophase is recorded (“yes” if it was occurring, and “no” if it was not; [29]). In this analysis, we evaluated the date of first yes (i.e., the first day of year [DOY] in which a phenophase had a positive observation) for four phenophases, “breaking leaf buds,” “leaves,” “open flowers,” and “ripe fruits.” We accessed the “site phenometric” data type [30], which returns the mean date of first “yes” for all individuals of the species that are monitored at a site. If only a single individual of a species is monitored at a site, the values for this individual are used. We chose the “breaking leaf buds,” “leaves,” “open flowers,” and “ripe fruits” phenophases because they best represent the full expression of a state in a plant and are included in > 94% of the USA-NPN’s species-specific plant phenology protocols [29].

### Thermal-time models

We constructed species and phenophase-specific thermal time models, which use growing degree days as forcing units based on a fixed start date to predict the date of a phenophase transition [2, 31, 32]. For each of these models, the phenology observations from the NPDb served as the inputs, and accumulated temperature thresholds were the intended output. We extracted site phenometric records for all possible combinations of species × phenophase (for “breaking leaf buds,” “leaves,” “open flowers,” and “ripe fruits”) from the NPDb on September 25, 2016 using the USA-NPN’s application program interface (API) for the period January 1, 2009 through September 25, 2016 [30]. We retained the first “yes” record following Jan 1 for a species × phenophase at a site. Observations for 2009–2015 were used to construct models, and observations from 2016 were used for model validation. We followed this approach for splitting data into calibration and validation pools, rather than a random 50–50 split or other split, to maximize sample sizes for model construction.

We trimmed the dataset to retain records where the number of days between the mean first “yes” for a phenophase and the mean prior “no” was < 15 days. Gerst et al. [33] demonstrate that in instances when site-level information is important, as in the present study, data users should cautiously constrain the temporal window between the prior “no” and the first “yes.” We chose 15 days as a threshold in an attempt to maximize sample size while constraining the error in onset date, represented as the temporal window between the prior “no” and the first “yes.”

We excluded records with days of first “yes” occurring after particular cut-offs to minimize the influence of outliers: for the “breaking leaf buds” and “leaves” phenophases, this cut-off was DOY 172 (June 21, the first day of summer, after which we expected to see few onsets of breaking leaf buds and leaves); for the “open flowers,” and “ripe fruits” phenophases, this cut-off was DOY 213 (Aug 1, a date after which we expected to see few onsets of open flowers and ripe fruits). We required a minimum of 30 site × year combinations for each analysis. Finally, we excluded the few records for Alaska because of the lack of climate data for these regions.

We constructed universal accumulated growing degree day (AGDD) thresholds for each species × phenophase independently. We define a universal AGDD threshold as the AGDD value associated with the onset DOY of a phenophase independent of year or site location. AGDD was determined by the accumulated heat units (0°C base temperature) from January 1^st^ to the first reported “yes” DOY for the phenophase at each sampling location and for each year.

We used PRISM daily gridded temperature data time-series [34, 35] (4km spatial resolution) based on site latitude and longitude to determine daily heat units. This yielded an AGDD value for each site × year record for each species × phenophase.

To calculate thresholds for each species × phenophase, we averaged the derived the AGDD values across site × years (2009–2015). Next, for each species × phenophase, we determined the actual DOY the AGDD threshold was met for each site × year (2009–2016) by repeating the calculation of AGDD at each location starting on January 1^st^ and returning the DOY the threshold was met. This allowed us to evaluate how well the candidate thresholds performed at predicting the DOY for calibration data points (2009–2015) as well for independent validation data points (2016). All model construction and evaluation was performed in R [36].

### Null models

For each species × phenophase combination, we constructed null models by calculating the mean of the mean DOY of first reported “yes” across site × years for the period 2009–2015. This value was then used to predict the phenophase transition for each year by determining the DOY the threshold value was met in each site × year. This predicted DOY was differenced from the actual observed DOY to assess the performance of this model. These null models served as a basis for comparing the performance of our AGDD models.

### Evaluating model performance

To evaluate model performance, we calculated mean absolute error (MAE), root mean squared error (RMSE), Nash-Sutcliffe model efficiency coefficient (ME) [37], and R^*2*^ for all species × phenophase models for both the 2009–2015 development dataset and the 2016 validation dataset. MAE and RMSE describe the difference between modeled and observed phenophase dates for each species × phenophase combination. ME compares the performance of the AGDD threshold models with that of the null models; ME can range from −∞ to 1, where 1 is a perfect match of modeled to observed data. A positive ME indicates that the AGDD model performs better than the null model for a species × phenophase, meaning the modeled threshold yielded fewer days between the predicted and reported DOY of onset compared to the null model. We established minimum criteria for further consideration of candidate models of ME ≥ 0.4 [25], R^*2*^ ≥ 0.5, and MAE ≤ 10 days. An MAE of 10 days was selected because it is consistent with other similar, recent studies [13, 16]. For each site × year for each candidate model, we differenced the predicted and reported day of onset for both the 2009–2015 and 2016 datasets.

### Evaluating model geographic extensibility

For the models that met minimum criteria for model fit and error, and for those species with readily available distributional range data, we determined how representative the model was across the range of the species using the 2009–2015 dataset. Our logic was to extend the model to portions of the species range with temperature conditions represented by the locations—or calibration points—used to construct the models.

We used mean spring temperature for this evaluation because it is a strong proxy for the continuously increasing AGDD variable used in the models. Spring temperatures were calculated by averaging the long-term (1981–2010) mean January, February, March, and April (JFMA) temperature layers [34]. We extracted the spring temperature values for the calibration points from this gridded layer using ArcGIS 10.1. Next, for each species × phenophase combination, we clipped this spring temperature grid using a shapefile of the species’ geographic distribution [38]. Finally, for each species × phenophase model, we determined the portion of the species range to which the model could be extended by limiting the range to locations with spring temperatures falling within the climate envelope of the calibration points.

### Demonstrating the use of models in short-term forecast map production

Thermal time models that emerge through this effort could be used by USA-NPN to produce daily and short-term forecast maps predicting the timing of phenophase transitions several days into the future on a nightly basis using available gridded temperature forecasts. We demonstrate this process using one of the candidate models that emerged through this study: the model for *Hamamelis virginiana*–leaves. We generated a map to show the DOY that the threshold for this species × phenophase was met in 2016 at all locations across the continental United States by calculating AGDDs from January 1^st^ and returning the DOY the threshold was met. Next, we clipped this map to the species distribution, as dates of a phenophase transition for locations outside of a species distribution are meaningless. Finally, we further clipped the map to the proportion of the species’ range that fell within the climate envelope of the calibration points as a way of characterizing the model’s geographic extensibility.

## Results

### Accumulated growing degree day models

There were sufficient observations in the NPDb to construct models for 107 species × phenophase combinations. The data used in constructing these 107 models totaled 10,604 unique site × species × year locations. Of these 107 models, 26 (24%) exhibited ME values ≥ 0.4, indicating that the threshold model performed better than the null model. We constrained this set of models by applying our minimum criteria for R^*2*^ and MAE, resulting in 15 candidate models that met all minimum criteria: one representing the “breaking leaf buds” phenophase, 7 representing “leaves,” and 7 representing “open flowers” (Table 1). Details for species × phenophase models that did not meet these criteria are presented in S1 Table.

**Table 1:** Details pertaining to candidate thermal time species × phenophase models that emerged from an evaluation of data maintained by the USA National Phenology Network, including mean absolute error (MAE), root mean square error (RMSE), and Nash-Sutcliffe model efficiency (NSME).

Species	Pheno-phase	Growth habit	2009–15 n	MAE Null model	RMSE Null model	2009–15 MAE	2009–15 RMSE	2009–15 r^2^	NSME	2016 n	2016 MAE	2016 RMSE	2016 r^2^	% of range model extends to	AGDD Threshold
*Amelanchier canadensis* (Canadian serviceberry)	breaking leaf buds	shrub/ tree	33	12.4	15.1	7.3	9.1	0.71	0.64	4	10.8	12.9	0.52	N/A	454
*Forsythia* spp. (forsythia)	leaves	shrub	149	14.9	17.6	7.0	8.8	0.80	0.75	19	5.9	8.6	0.59	N/A	681
*Amelanchier canadensis* (Canadian serviceberry)	leaves	shrub/ tree	37	12.5	15.4	3.9	4.5	0.92	0.91	4	7.5	7.9	0.86	N/A	646
*Cercis canadensis* (eastern redbud)	leaves	shrub/ tree	92	15.4	19.5	9.3	12.3	0.74	0.60	22	7.8	10.1	0.92	99.8%	1,132
*Cornus florida* (flowering dogwood)	leaves	shrub/ tree	178	12.5	15.8	9.3	11.9	0.65	0.43	42	10.5	13.6	0.73	93.9%	985
*Hamamelis virginiana* (American witchhazel)	leaves	shrub/ tree	33	9.1	12.0	6.6	9.3	0.63	0.40	13	14.1	15.7	0.80	49.8%	737
*Betula papyrifera* (paper birch)	leaves	tree	79	8.6	11.3	6.4	7.9	0.71	0.52	15	6.0	9.0	0.55	93.3%	655
*Carya glabra* (pignut hickory)	leaves	tree	31	13.5	19.1	8.4	11.8	0.73	0.62	6	9.3	11.6	0.65	96.1%	993
*Maianthemum canadense* (Canada mayflower)	open flowers	forb/herb	56	11.8	15.0	8.1	10.2	0.58	0.53	26	9.2	12.2	0.33	N/A	1,040
*Forsythia* spp. (forsythia)	open flowers	shrub	137	16.8	20.1	6.5	8.8	0.83	0.81	19	8.9	12.8	0.92	N/A	458
*Vaccinium corymbosum* (highbush blueberry)	open flowers	shrub	36	26.0	29.4	8.2	12.5	0.82	0.82	5	6.6	9.1	0.74	N/A	891
*Amelanchier canadensis* (Canadian serviceberry)	open flowers	shrub/ tree	35	12.7	15.7	3.5	4.5	0.92	0.92	4	5.3	6.2	0.42	N/A	651
*Prunus serotina* (black cherry)	open flowers	shrub/ tree	85	18.8	26.4	9.0	11.2	0.85	0.82	22	18.3	21.5	0.83	97.2%	1,300
*Betula papyrifera* (paper birch)	open flowers	tree	32	8.7	10.4	5.8	7.5	0.75	0.48	6	15.2	20.8	0.80	89.0%	571
*Quercus alba* (white oak)	open flowers	tree	48	15.0	17.7	7.5	9.9	0.80	0.69	18	11.9	15.6	0.22	88.3%	948

**Fig 1:**
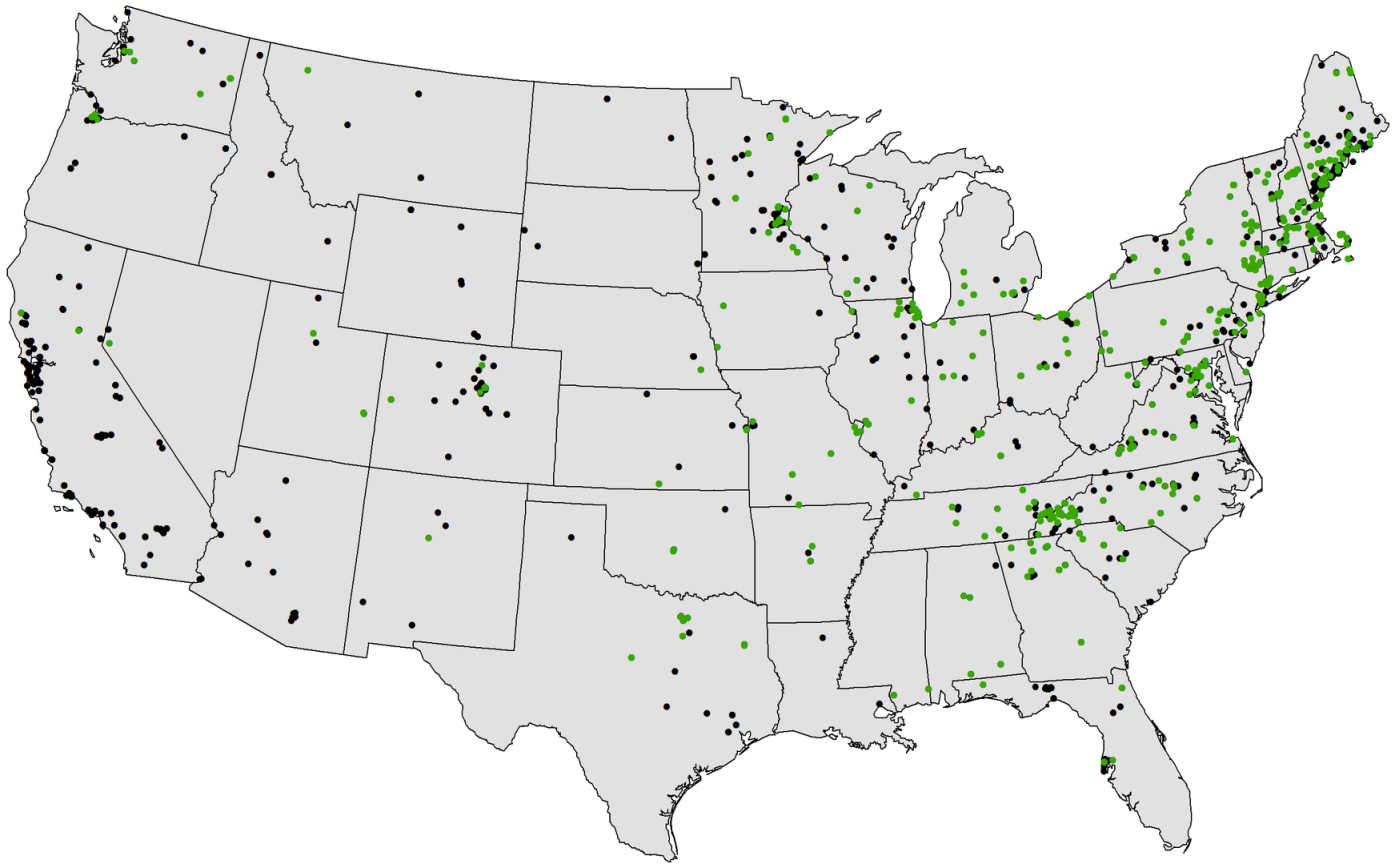
Geographic distribution of species *×* year data points used in constructing 107 unique species *×* phenophase models. Black dots represent data points for all models (n = 10,604); green dots represent points used in constructing the 15 candidate models (n = 1,893).

Fourteen of the 15 candidate models were for plants with the shrub, tree, or shrub/tree growth form, whereas one model represented non-woody growth form, based on the USDA PLANTS database [39] (Table 1). “Open flowers” was the only phenophase with high-performing models across all growth forms (S1 Fig). The mean (± SD) for the 15 candidate models was 7.1 ± 1.8 days and 0.76 ± 0.10 for MAE and R^2^, respectively. Sites used in model construction, a total of 10,604 unique site × species × year locations, were spread across the continental United States, with greater concentration in the eastern U.S. (Fig 1). The dataset used in constructing the 15 candidate models was comprised of 1,893 unique site × species × year locations, and were similarly spread across the continental United States, with greater concentration in the eastern U.S. (Fig 1).

Accumulated GDD thresholds that emerged for the candidate species × phenophases evaluated ranged from 454 GDDs (*Amelanchier canadensis*-breaking leaf buds) to 1,300 GDDs (*Prunus serotina*-open flowers, Table 1).

When evaluated against the independent 2016 validation points, the mean (± SD) for the 15 candidate models was 9.82 ± 3.74 days and 0.66 ± 0.21 for MAE and R^2^, respectively (Table 1), although some models (e.g., *Amelanchier canadensis*-leaves, *Forsythia* spp.-open flowers) exhibited nearly identical values when run with development and validation datasets.

**Fig 2:**
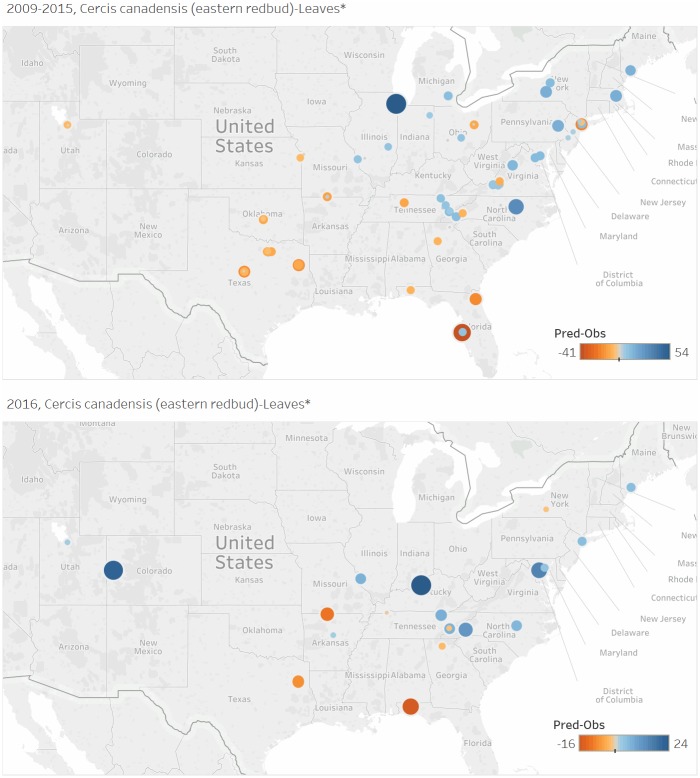
Observation locations used to a) construct and b) test the thermal time model for *Cercis canadensis*-leaves. Point size and color represent the difference, in days, between the predicted and the observed day of year for leaf-out. Locations where the model predicted leaves earlier than observer reports are shown in orange; locations where the model predicted leaves later than observer reports are shown in blue.

In all candidate species × phenophase models, the difference between the predicted versus observed onset DOY value varied by site. Maps depicting the predicted-observed days for development (2009–2015) and validation (2016) data points for the 15 candidate models appear in S1 Appendix. The predicted-observed onset DOY show a geographic pattern in several of the candidate species × phenophase models. For example, the *Cercis canadensis*–leaves model predicts the onset of the “leaves” phenophase too late in nearly all northern sites and too early in nearly all southern sites (Fig 2). This pattern is also apparent in *Cornus florida*-leaves, *Forsythia* spp.-open flowers, *Forsythia* spp.-leaves, and *Prunus serotina*-open flowers (S1 Fig).

### Geographic extensibility

**Fig 3:**
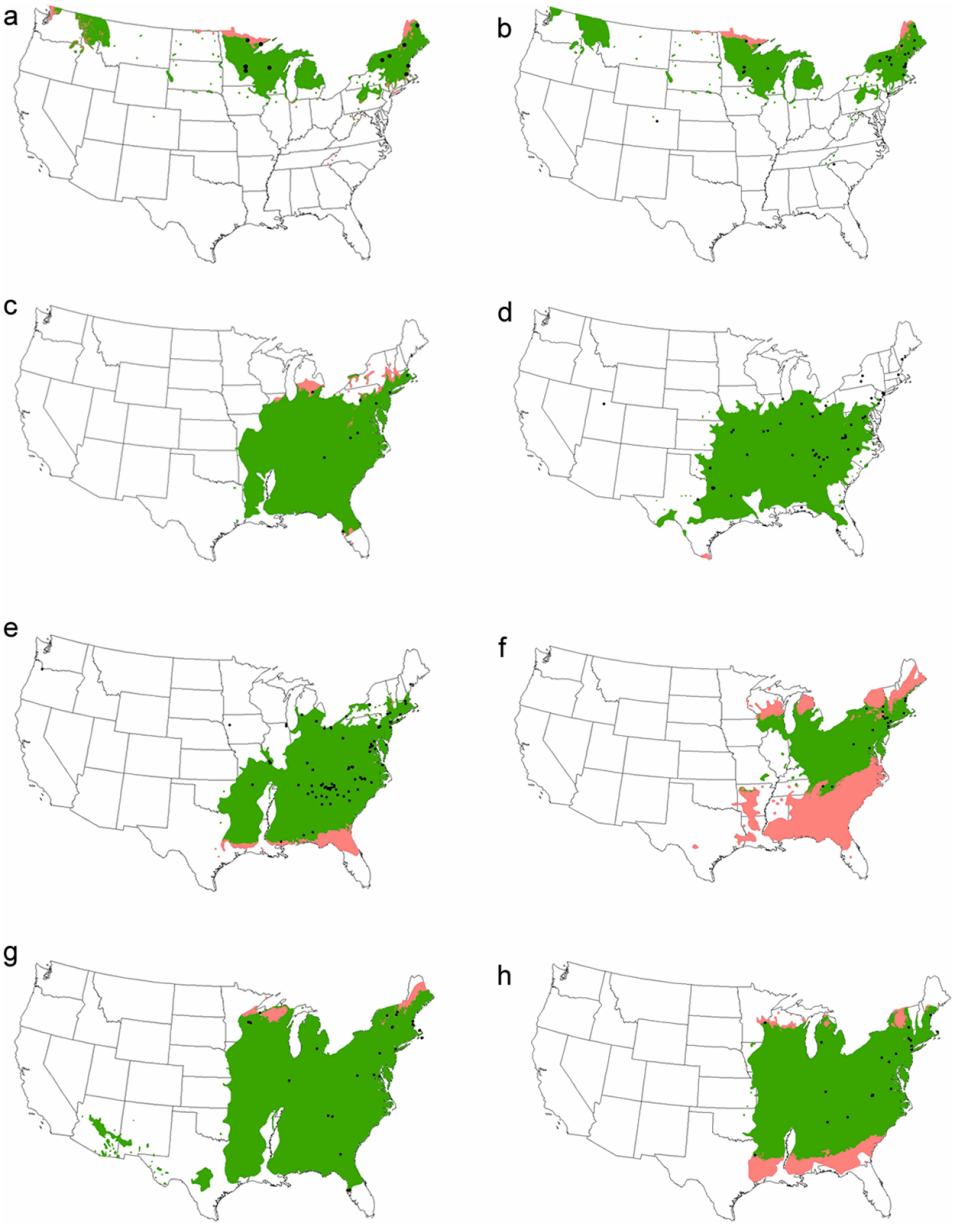
Estimates of geographic extensibility for eight species × phenophase thermal time models. a) *Betula papyfera*–open flowers; b) *Betula papyfera*–leaves; c) *Carya glabra*–leaves; d) *Cercis canadensis*-leaves; e) *Cornus florida*–leaves; f) *Hamamelis virginiana-*leaves; g) *Prunus serotina*–open flowers; h) *Quercus alba*–open flowers. The known species range within the US is represented by the shaded polygon; the green polygon represents the portion of the range represented by the model. Black points represent observation locations used to construct the models.

Species range maps were available for only 8 of the 15 candidate models. The proportion of the species range to which models could be extended ranged from 50% (*Hamamelis virginiana*–leaves) to 99.8% (*Cercis canadensis*–leaves; Table 1, Fig 3). For the species where the models did not extend to the majority of the species range, such as *Hamamelis virginiana*-leaves and *Quercus alba–*open flowers (Fig 3), the portions of the range that were excluded from the model extensibility were the extreme northern and southern regions.

### Generating forecast maps with candidate models

**Fig 4:**
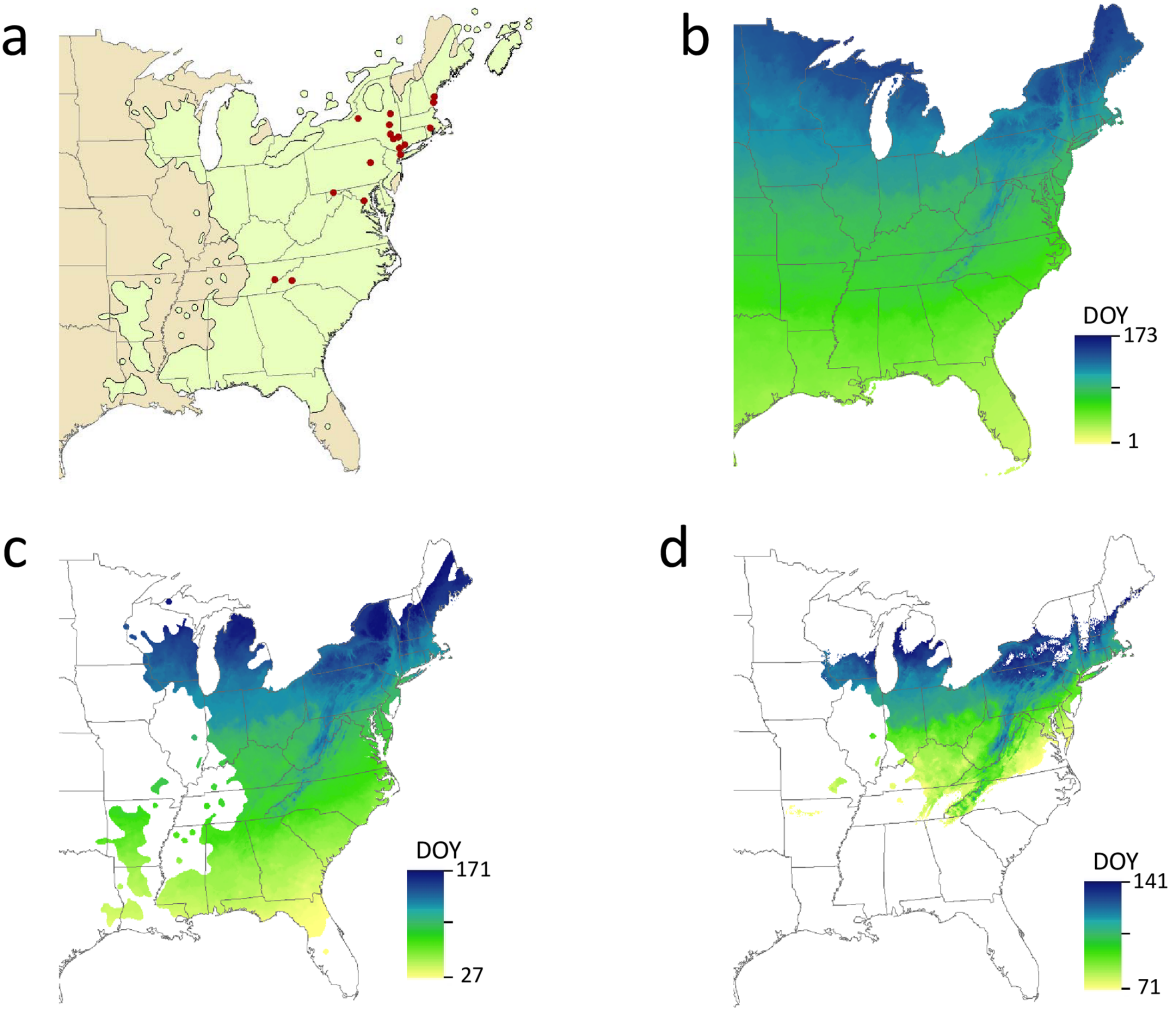
Proposed production workflow for the *Hamamelis virginiana*–leaves model to generate daily maps and short-term forecasts of leaf-out. a) Calibration points used to construct the model overlayed on *Hamamelis virginiana* range (Little 1999); b) model run on 2016 daily temperature data to show day of year the AGDD threshold was met; c) model results clipped to the distribution of *Hamamelis virginiana*; and d) the distribution further clipped to the proportion of the species’ range that fell within the climate envelope of the calibration points.

An example of how the candidate models that emerged through this effort could be put into production to generate daily and short-term forecast maps predicting the timing of phenophase transitions several days into the future on a nightly basis is presented in Fig 4. Across the entire continental U.S., the DOY the threshold for *Hamamelis virginiana*–leaves was met ranged from Jan 1 (DOY 1) to Jun 22 (DOY 173; Fig 4b). Within the species range, the DOY this threshold was met ranged from Jan 27 (DOY 27) to Jun 20 (DOY 171; Fig 4c); once this map was further clipped to the proportion of the species’ range that fell within the climate envelope of the calibration points, the DOY this threshold was met ranged from Mar 12 (DOY 71) to May 21 (DOY 141; Fig 4d).

## Discussion

The aim of this study was to explore the potential that exists in the broad and rich volunteer-collected dataset maintained by the USA-NPN for constructing models predicting the timing of phenological transition across large geographic regions. Remarkably, given the limited temporal depth of this dataset and the simple modeling approach used, this effort resulted in several models that performed well across much of the known species distributions. Of the 15 candidate models that emerged, the majority represented the “leaves” phenophase and the shrubs and trees growth habit (Table 1, S1 Fig), similar to recent studies using data maintained by the USA-NPN [9–13]. Another large portion (47%) of the candidate models that emerged from this analysis represented the “open flowers” phenophase. A single candidate model, *Maianthemum canadense*-open flowers, represented the forb/herb growth form. Our ability to develop high-performing models with a simple thermal time approach suggests that the data in the NPDb hold promise for developing and enhancing models predicting phenological transitions, beyond what is accomplished in this simple approach. Further, these models have immediate utility, as they can be used in a wide range of management applications.

For a small number of the species **×** phenophase transitions for which candidate models emerged in this study, models have been published previously. For example, both Melaas et al. [13] and Jeong et al. [11] established predictive models of leaf phenophases for *Betula papyfera* using slightly more complex formulations; RMSE values reported for these models varied from those in the present study by 1–2 days. Herms [40] report accumulated temperature thresholds for “first bloom” of two species of *Forsythia* and *Prunus serotina* based on a 50°F base temperature. To our knowledge, the thermal time thresholds that emerged in this study are the first predictive phenology models for several of the species **×** phenophase transitions to be identified.

### Geographic extensibility

Whether a candidate model can be confidently utilized to predict the timing of phenological transition across a species’ entire geographic range is an important consideration for producing phenology maps. Several of our candidate models were developed using observations dispersed across wide latitudinal ranges. Encouragingly, the simple approach employed here demonstrates that for most of the candidate models, the samples of development points represent the range of conditions within the species’ range reasonably well. More specifically, for the 8 candidate models where we could explore geographic extensibility, the majority of the species’ range fell within the climate envelope of the calibration points. This finding underscores the utility of the threshold models that emerged from this study; maps generated using these thresholds appear to be viable across most of the species ranges.

### Applications for predictive phenology models

Though the focus of this effort was not to identify the exact trigger or set of triggers to phenological transitions, several high-performing models emerged that have the potential for use in a wide range of applications.

Predicting when a species will undergo a phenological transition, e.g., transitioning from closed flower buds to open flowers at a particular location, has value for a wide range of short-term natural resource management applications. For example, if the developmental status of a plant—such as whether buds have formed or broken yet—can be predicted across a region based on the accumulation of heat units, then local weather forecast offices can selectively issue frost warnings for upcoming cold events, broadcasting warnings to growers in regions where plants may be vulnerable [41]. Similarly, national-scale maps of leaf-out or flowering status for common horticultural plant species can be used by media outlets to warn homeowners of the risk of impending frost damage to particular species based on forecasted weather events [42]. Real-time and short-term forecast maps indicating the flowering status of particularly allergenic species can be used to anticipate regional allergy outbreaks and to warn allergy sufferers of especially problematic conditions [43, 44], and predictive maps of fruit ripening could be used to anticipate the timing of crops coming to market [45]. Finally, optimal timing of management activities such as pest, pathogen, and invasive species detection or treatment can benefit from real-time information and short-term forecasts of phenological transitions [4, 5].

The candidate AGDD threshold models developed in the present analysis could easily be folded into the USA-NPN’s existing workflow for producing daily and short-term forecast maps of accumulated growing degree days and the Extended Spring Indices for the United States [20], predicting the timing of phenophase transitions several days into the future on a nightly basis using available gridded temperature forecasts. We demonstrate this potential in Fig 4, which shows the DOY on which first leaves for *Hamamelis virginiana* are predicted for the portion of the species range in which the “leaves” model would be suitable.

Predictive maps generated using the models could also serve as a valuable way to further engage participants in *Nature’s Notebook*, the USA-NPN’s phenology observing program that populates the National Phenology Database. These additional incoming observations for particular species × phenophase combinations could then be folded into the development or validation datasets, leading to real-time model refinement. These incoming data points could be particularly valuable to fill in geographic regions or climate conditions not represented by existing data points, and thereby limiting model extensibility, as with *Hamamelis virginiana*-leaves (Fig 4).

### Opportunities for model improvement

Many (86%) of the species × phenophase combinations evaluated did not yield viable models (S1 Table). There are several possible reasons for this. First, the phenophase transitions for these species may not solely cued by temperature accumulated from a fixed January 1 start date; other variables not included in the models may be critical to improve model performance above our minimum thresholds for some species, including day length, frost events, specific weather events, or biotic conditions such as competition [46–49]. Second, local adaptation, or spatial unstationarity [49], may explain poor model fit in some species. Across geography, a species’ sensitivity to particular environmental cues to phenology can vary [18, 50, 51]. In the present study, we see evidence of local adaptation in *Cercis canadensis* when attempting to predict the start time for the “leaves” phenophase. Though the universal AGDD threshold performs well enough for this species × phenophase to qualify as a candidate model, there is clearly a latitudinal influence at play (Fig 2).

Finally, poor model performance could result from a lack of precision in the estimates of phenophase onset used in model construction. In an effort to maximize sample sizes in this model construction exercise, we allowed for up to 15 days between last reported “no” and first reported “yes” for the phenophase at a site. This temporal imprecision introduces additional non-climate related uncertainty around AGDD thresholds that negatively impacts model performance. Alternatively, model performance may be high, but the measurement error may result in poor model fit statistics. Imprecision in these estimates can be offset by large sample sizes: larger samples can buffer the effects of outliers and are more likely to capture more of the underlying landscape heterogeneity [33, 52]. However, for many of our species × phenophase combinations, our development samples were comprised of fewer than 100 points, and in nearly 30% of cases, fewer than 40 points (S1 Table). Accordingly, models for many species × phenophase combinations could have failed to emerge from this effort because of the combined effects of temporal imprecision and small sample sizes.

The issues of imprecise estimates of onset and small sample sizes in data curated by the USA-NPN is improving; observations of plant phenology are continuously being contributed to the NPDb by participants in *Nature’s Notebook* and also at dozens of U.S. National Ecological Observatory Network (NEON) sites across the U.S. [53]. However, a key insight from the present study is the large influence that imprecision around the onset dates in the data housed in the NPDb can have on the ability to generate high-performing models. Researchers using the data maintained by the USA-NPN for constructing predictive phenology models will want to bear this in mind.

Future efforts to construct predictive models using phenology observations maintained by the USA National Phenology Network could result in better performing models by incorporating a number of improvements, including an evaluation of different base temperatures [2, 32], adding a variable start date [13], or adding a photoperiod control, a chill requirement, and/or other abiotic variables. In addition, local adaptation could be accounted for either by constructing regional AGDD threshold models for some of these species (e.g., only locations above or below a certain latitude) or by using more sophisticated approaches to account for regional adaptation, as suggested by Liang [18].

#### Tradeoffs of sophisticated models

Applications requiring more precise spatially explicit estimates of phenophase transitions than the candidate models identified through this effort can offer may require more sophisticated modeling approaches. Such approaches could include statistical models incorporating a wide range of explanatory variables as well as process-based simulations. More complex model formulations may offer improvements in model performance, though a major tradeoff of adding variables and contingencies to predictive models of phenophase transition increases the complexity of producing and updating maps on a nightly basis following USA-NPN’s existing workflow. Further, more complex formulations can make real-time maps and short-term forecasts impossible, if variables included in the model are not available as gridded datasets and with the necessary immediacy. Finally, models incorporating multiple variables necessitate increased sample sizes.

## Conclusions

This research supports to our prediction that the data maintained by the USA-NPN holds promise for use in constructing models predicting the timing of phenological transition, as established in previous studies using limited sets of species [9–14]. This simple model-building effort sets the stage for the development of more sophisticated models predicting phenological transitions as the data housed in the NPDb grows. Further, this work demonstrates that in many cases models constructed using data currently available from the USA-NPN can be suitable for use across species’ ranges. Finally, this effort highlights the importance of additional phenology observations across space and time.

## Supporting information

S1 Table Species and phenophases for which there were sufficient data in the National Phenology Database to construct thermal time models of phenophase onset. * denotes candidate species × phenophase models that emerged; AGDD thresholds identified through this exercise are provided for these species × phenophases.
(XLSX)

S1 Fig Count of candidate thermal time species × phenophases models constructed using data housed in the National Phenology Data by phenophase and growth habit. Blue bars represent the proportion of models that did not meet criteria for ME, MAE, and R^2^; orange bars represent “candidate” models that did meet criteria.
(PDF)

S1 Appendix Maps of observation locations used to construct (top panel) and test (bottom panel) each of the species × phenophases thermal time models. Point size and color represent the difference, in days, between the predicted and the observed day of year for leaf-out. Locations where the model predicted leaves earlier than observer reports are shown in orange; locations where the model predicted leaves later than observer reports are shown in blue. Interactive maps available at https://tinyurl.com/usanpn-agdd-models.
(DOCX)
