# Supplementary figures and images for: USA National Phenology Network’s volunteer-contributed observations yield predictive models of phenological transitions
Source: PLoS One. 2017 Aug 22;12(8):e0182919. doi: 10.1371/journal.pone.0182919 (PMC5568737; doi:10.1371/journal.pone.0182919)

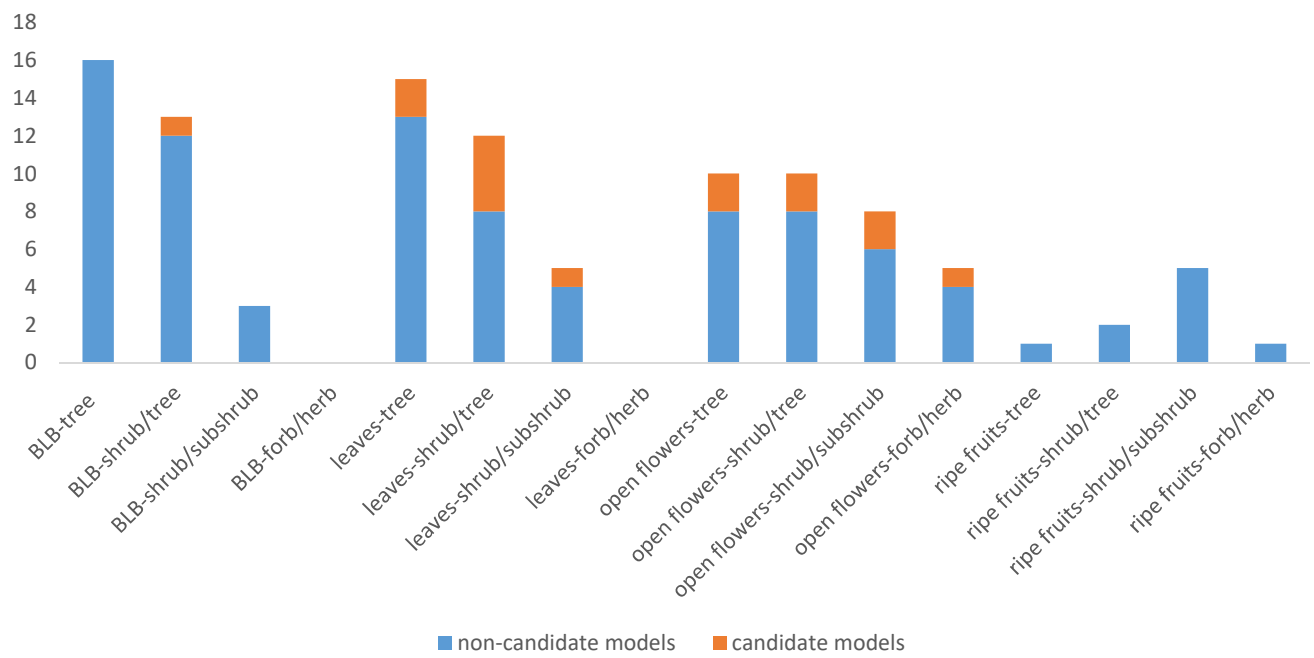

Supplement: S1 Fig — Blue bars represent the proportion of models that did not meet criteria for ME, MAE, and R2; orange bars represent “candidate” models that did meet criteria. (PDF) [file pone.0182919.s002.pdf]

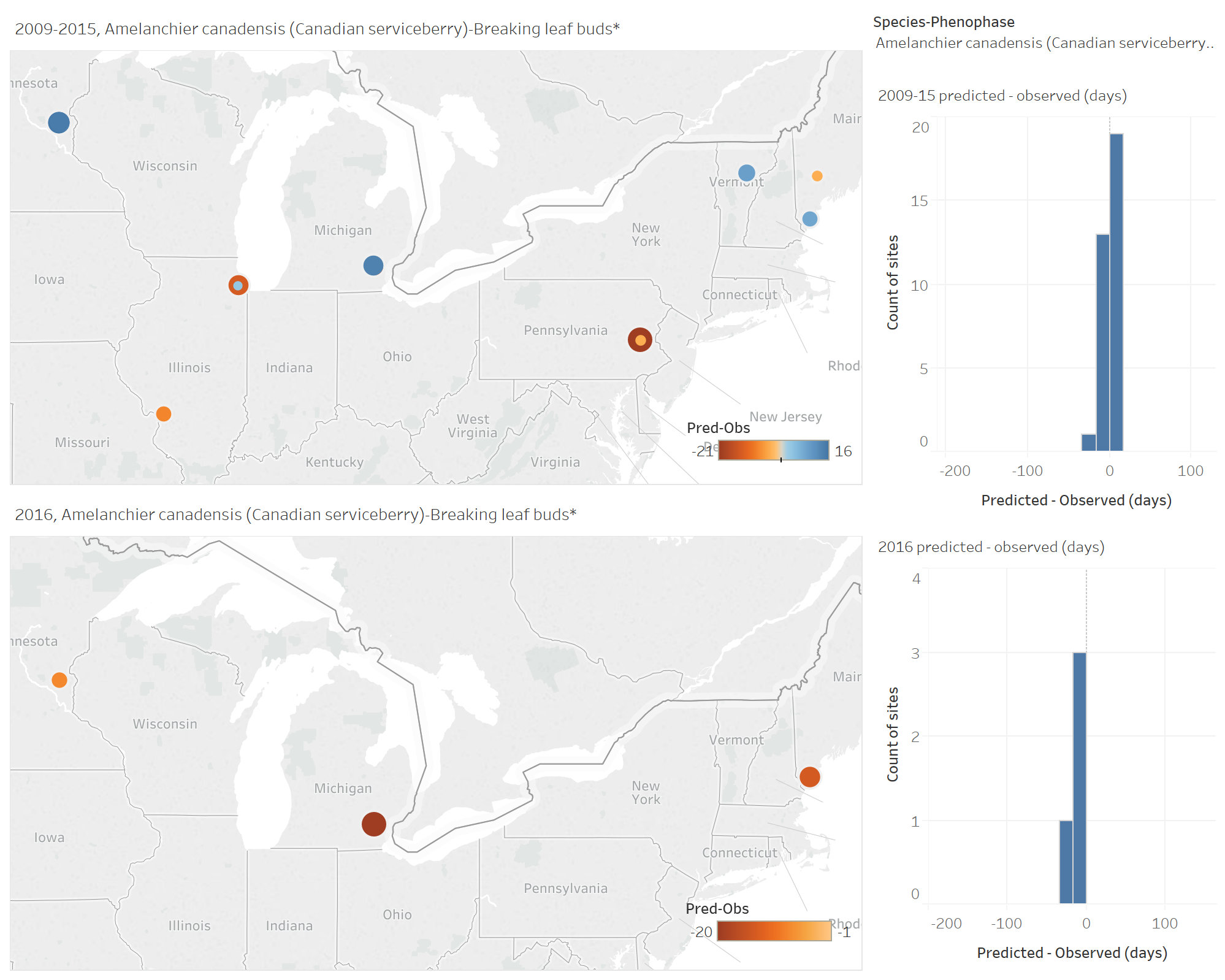

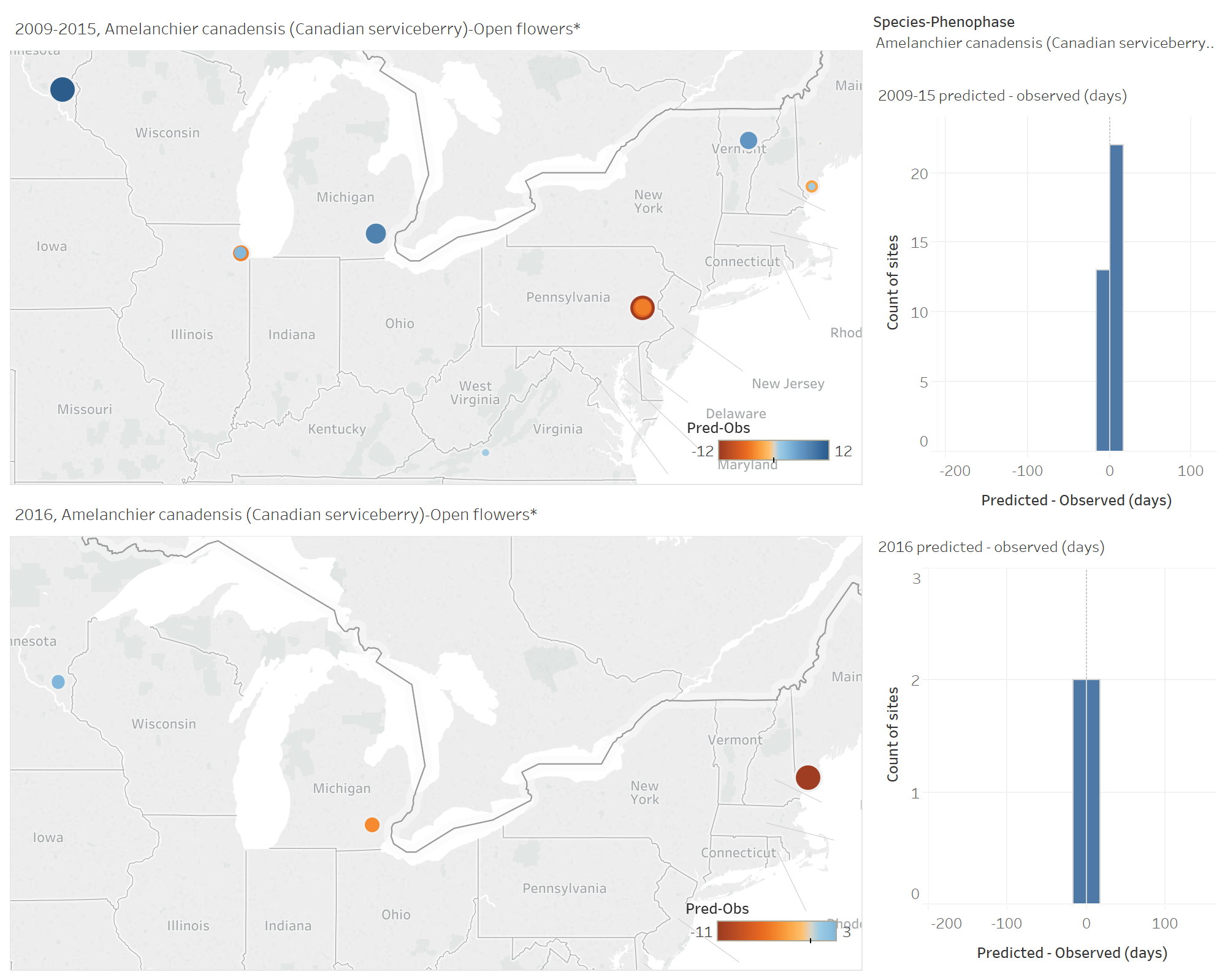

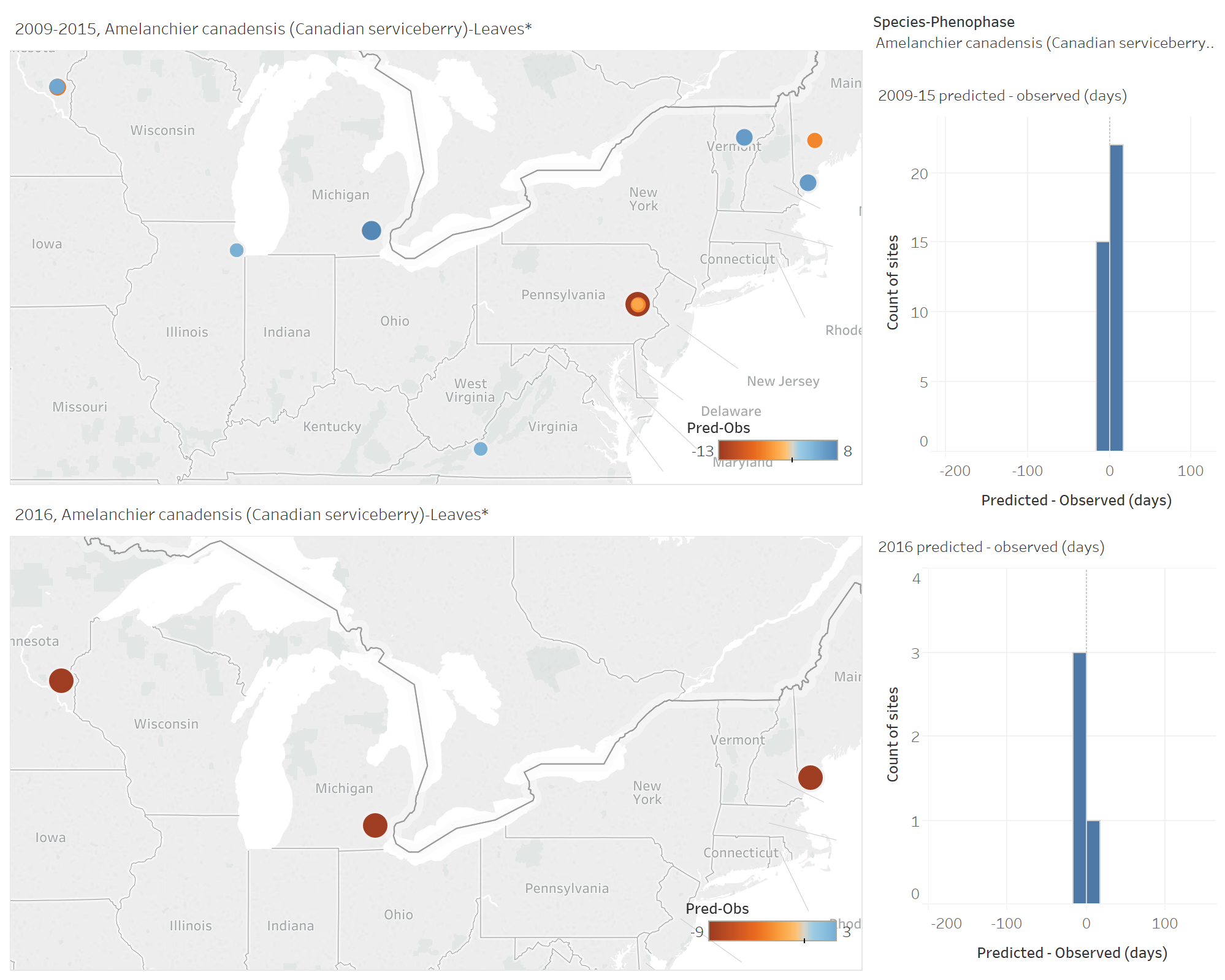

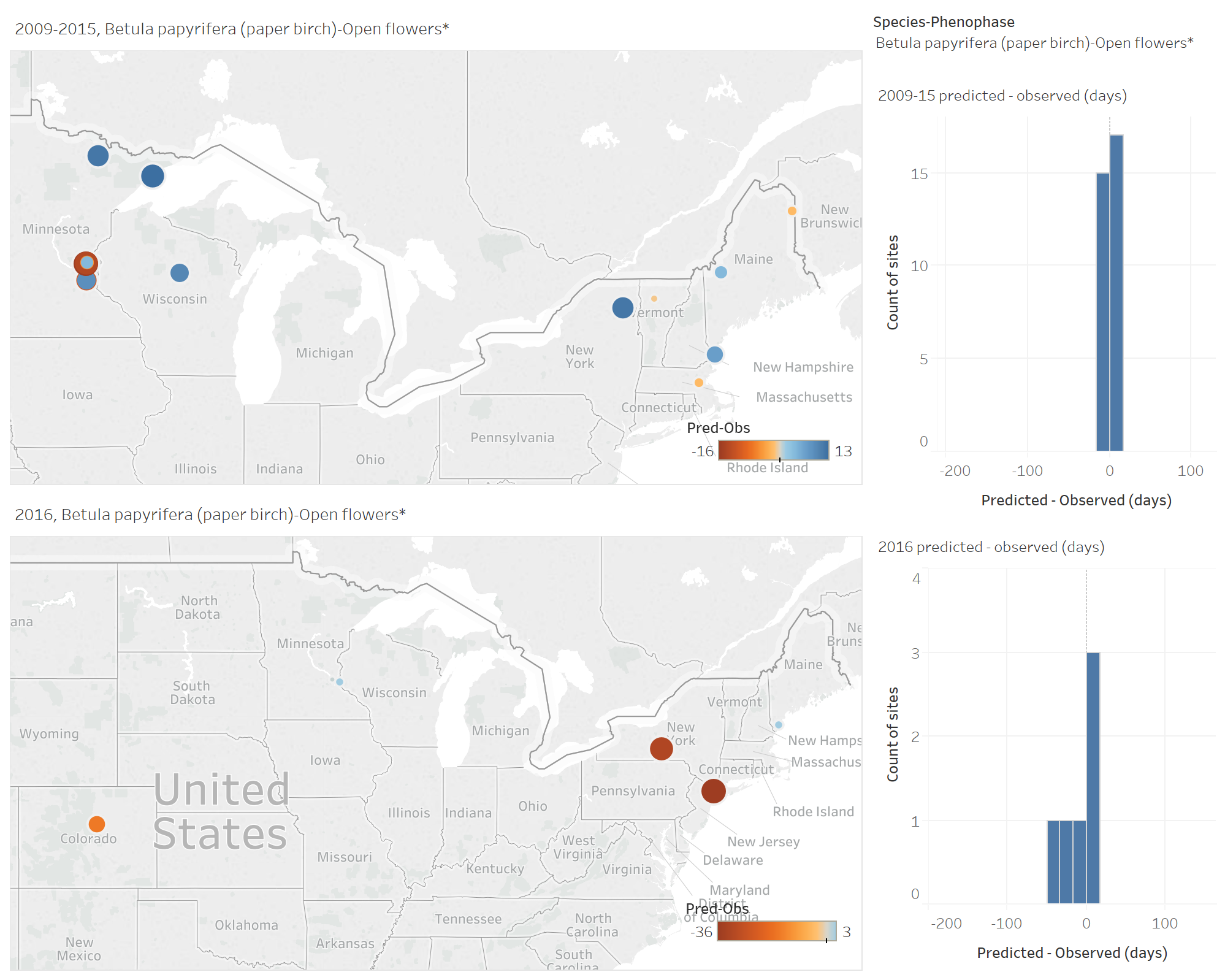

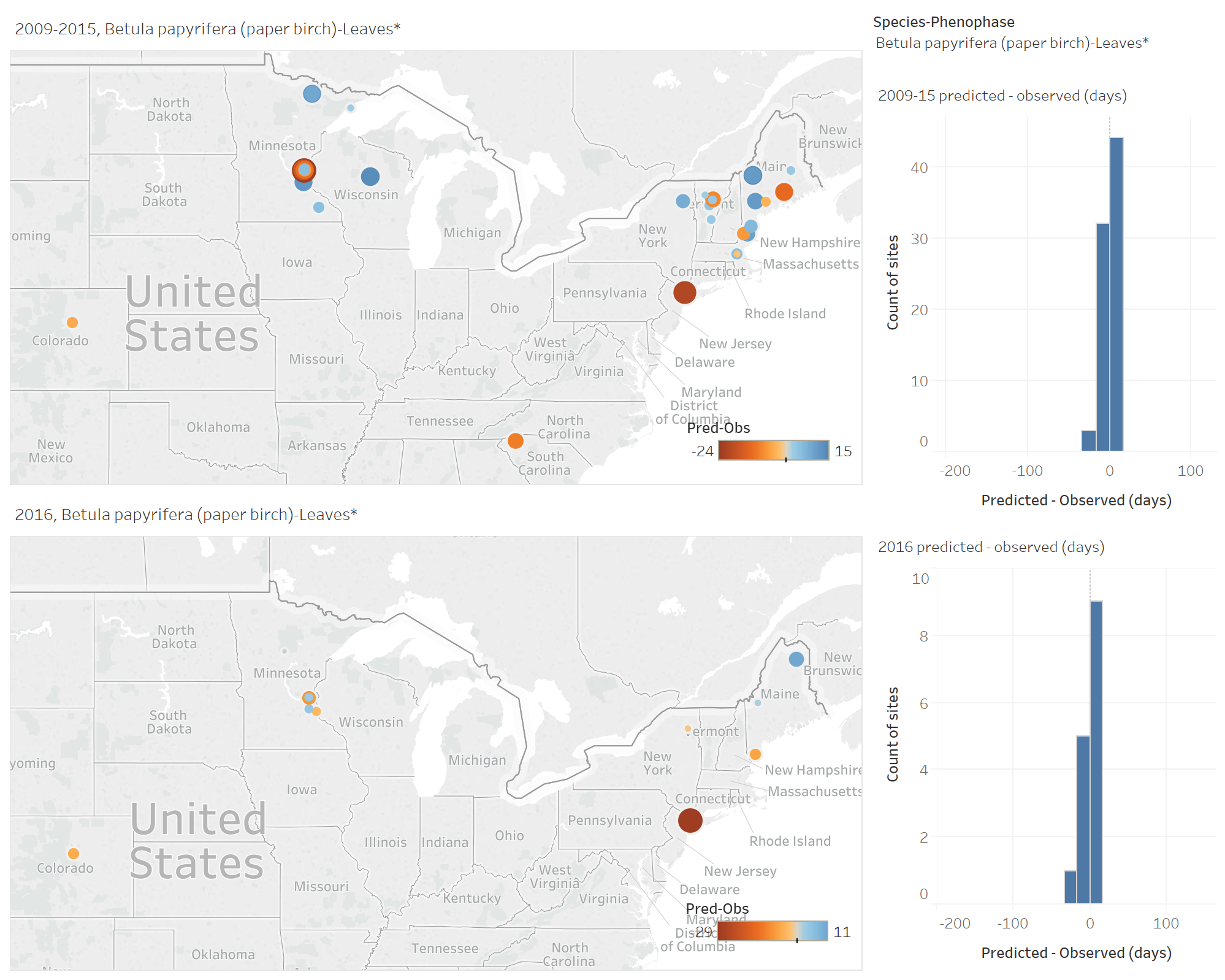


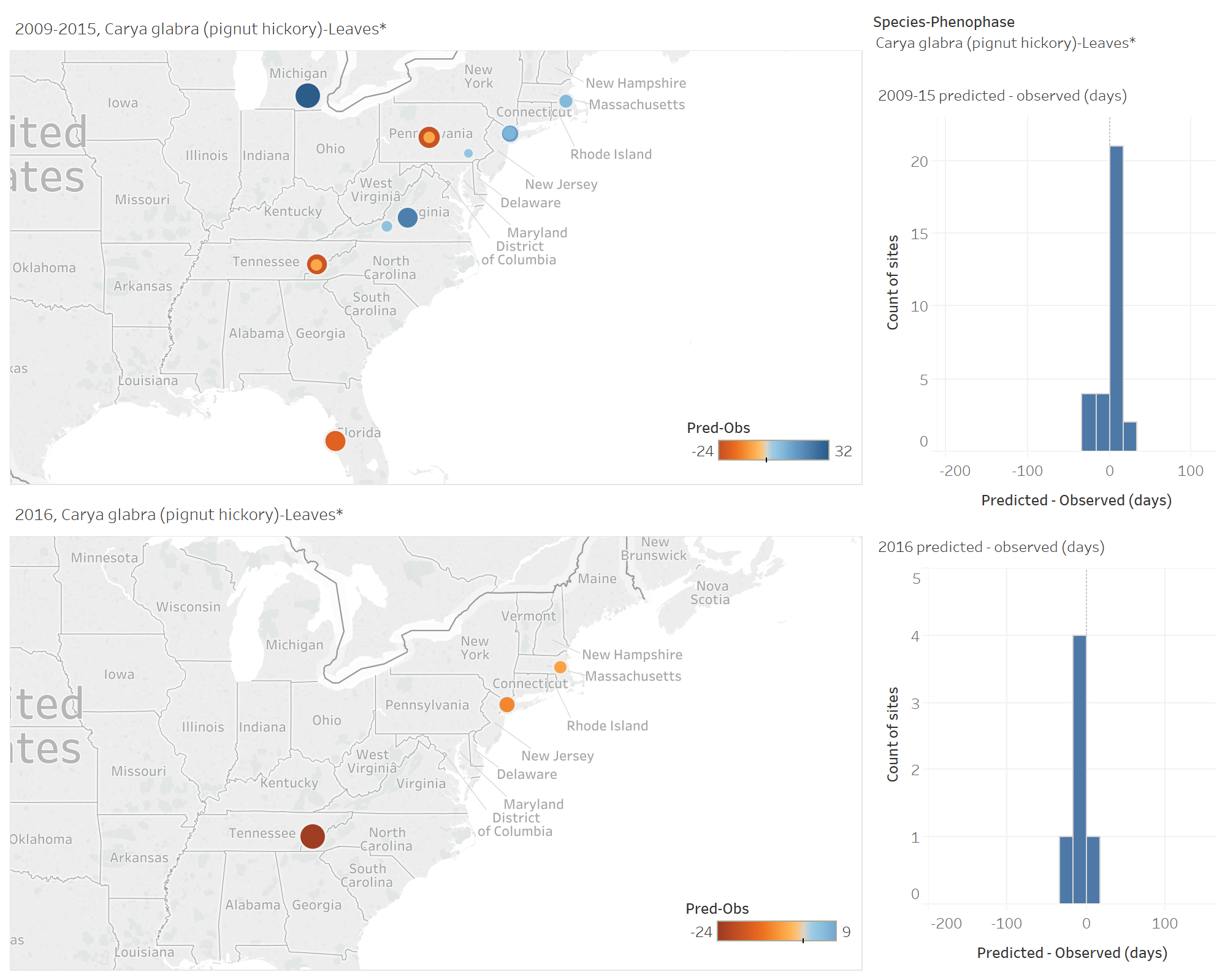

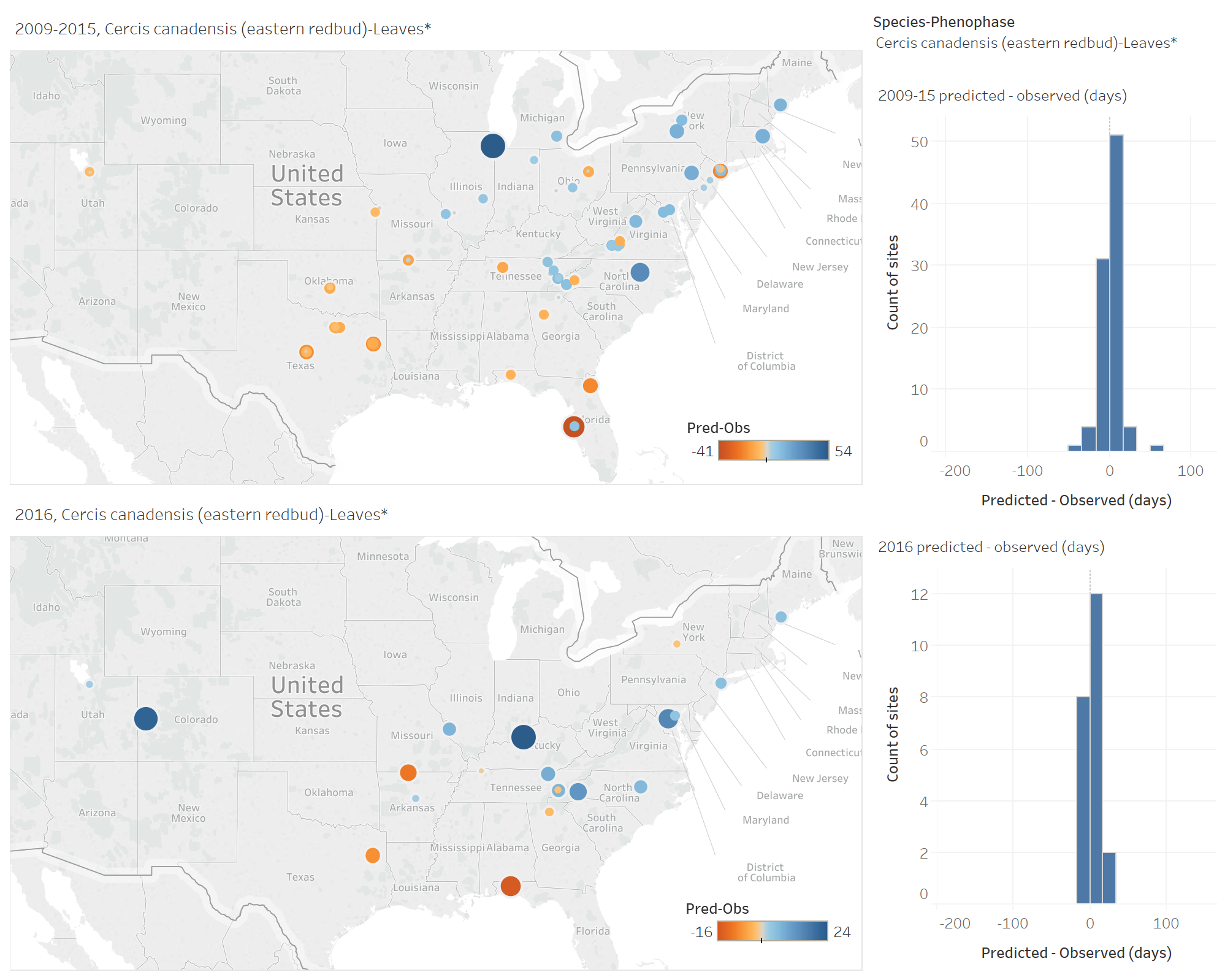


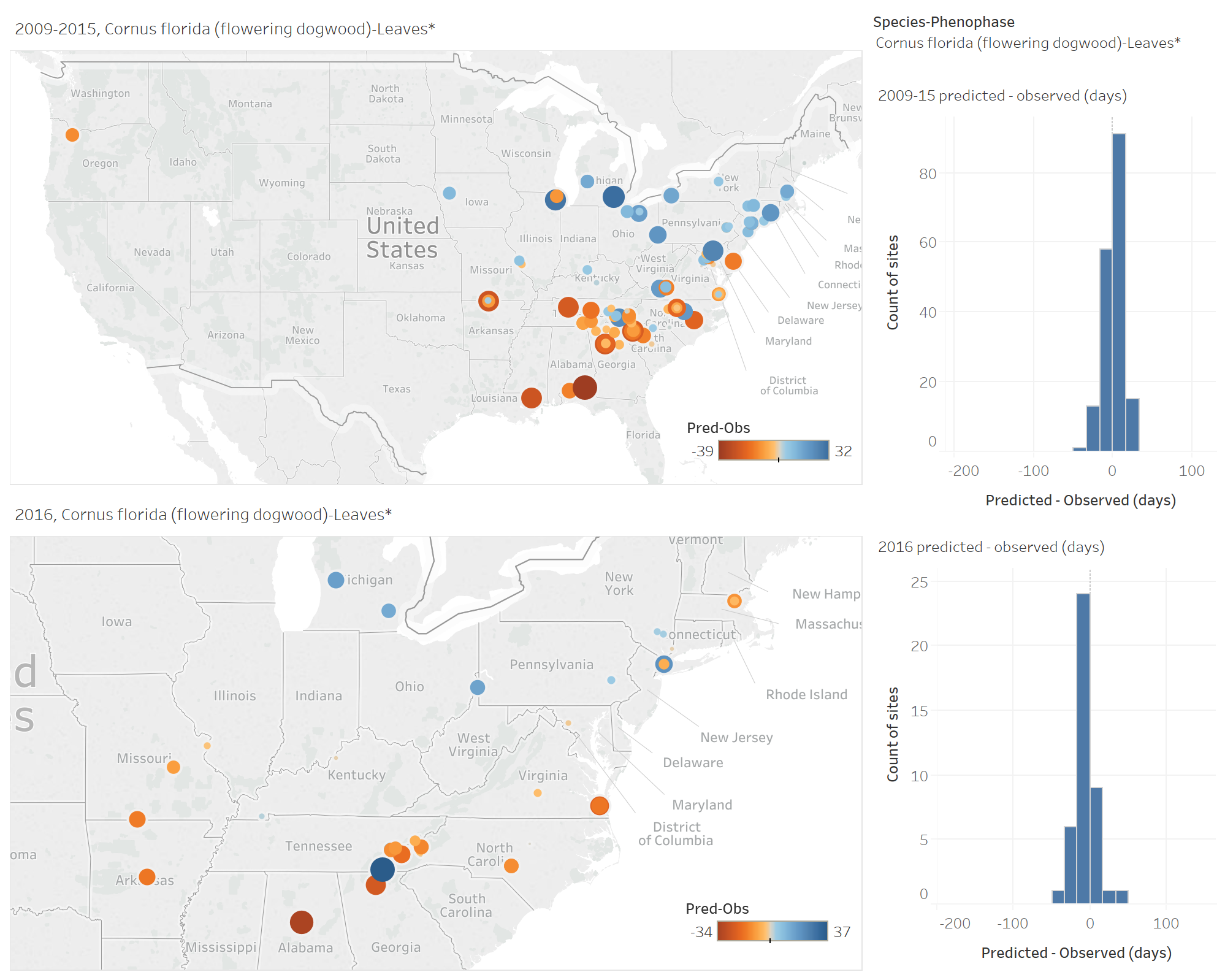

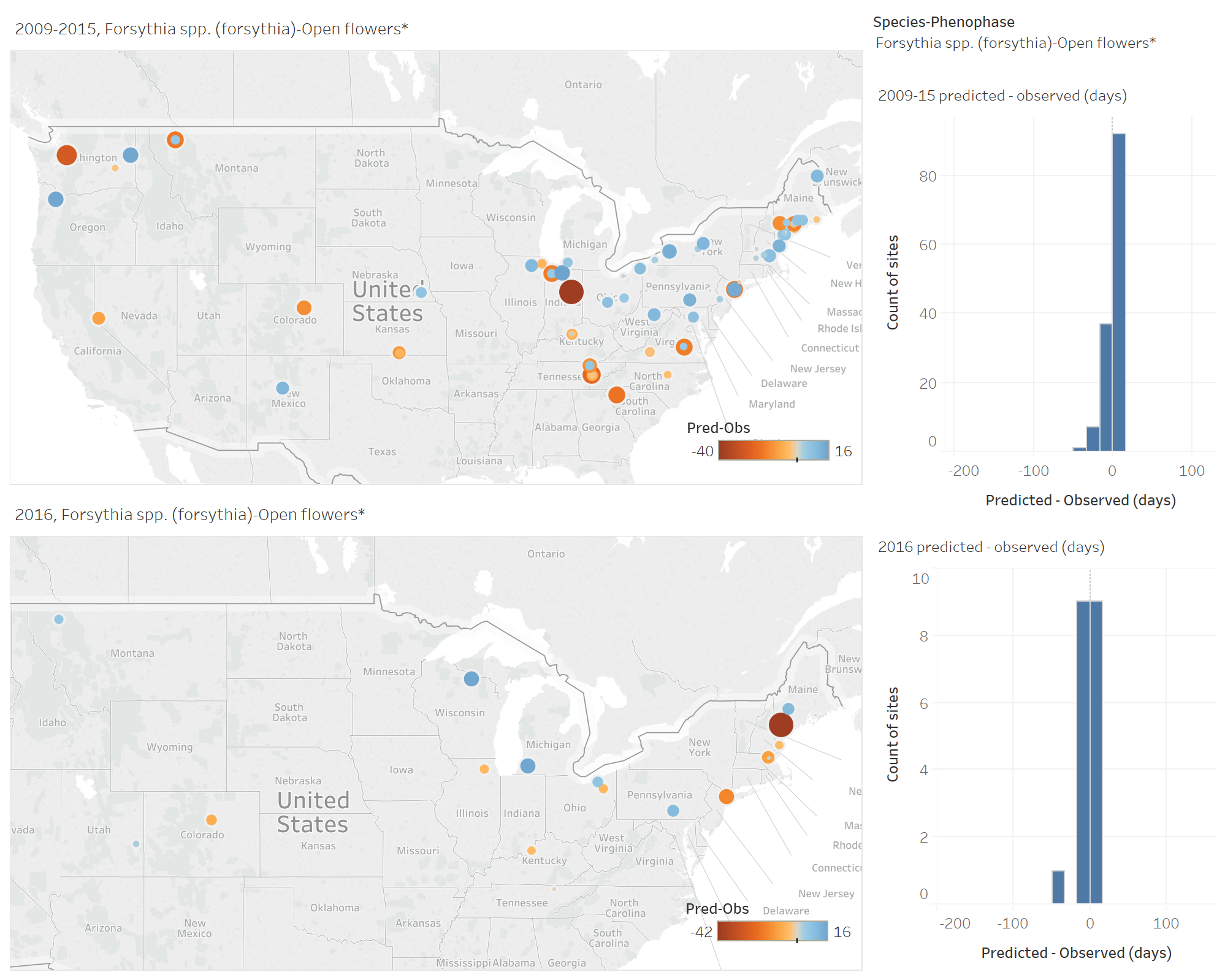

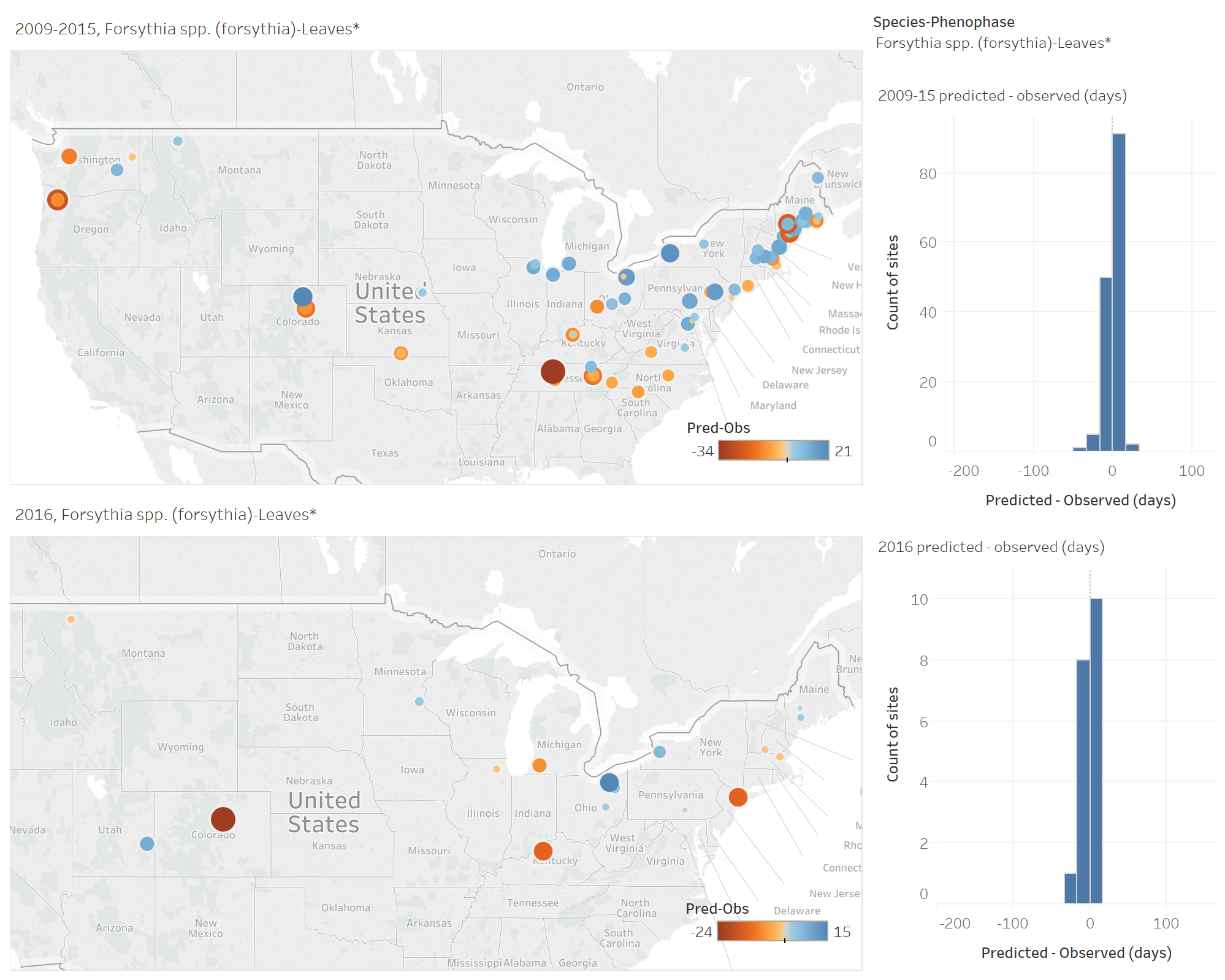


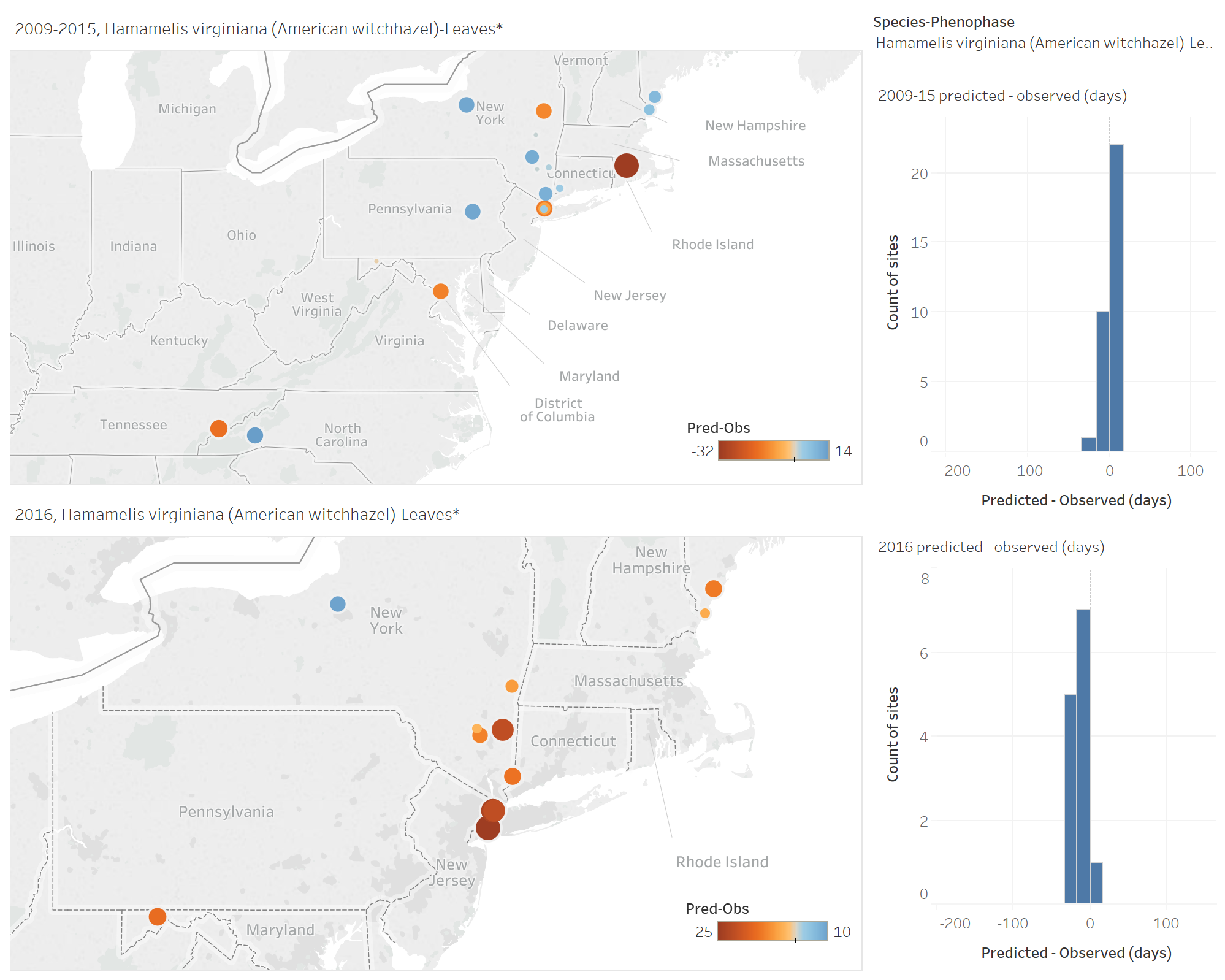

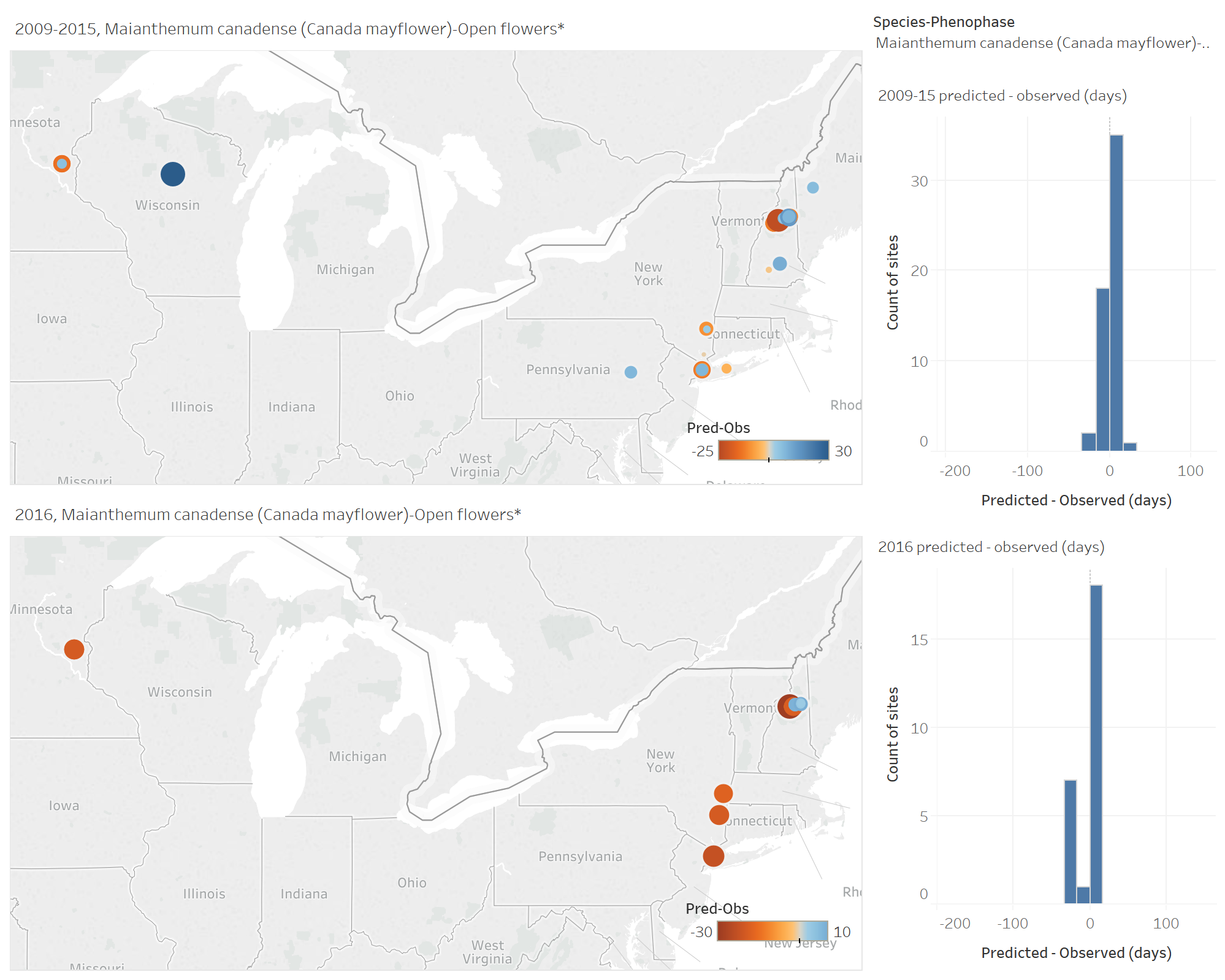

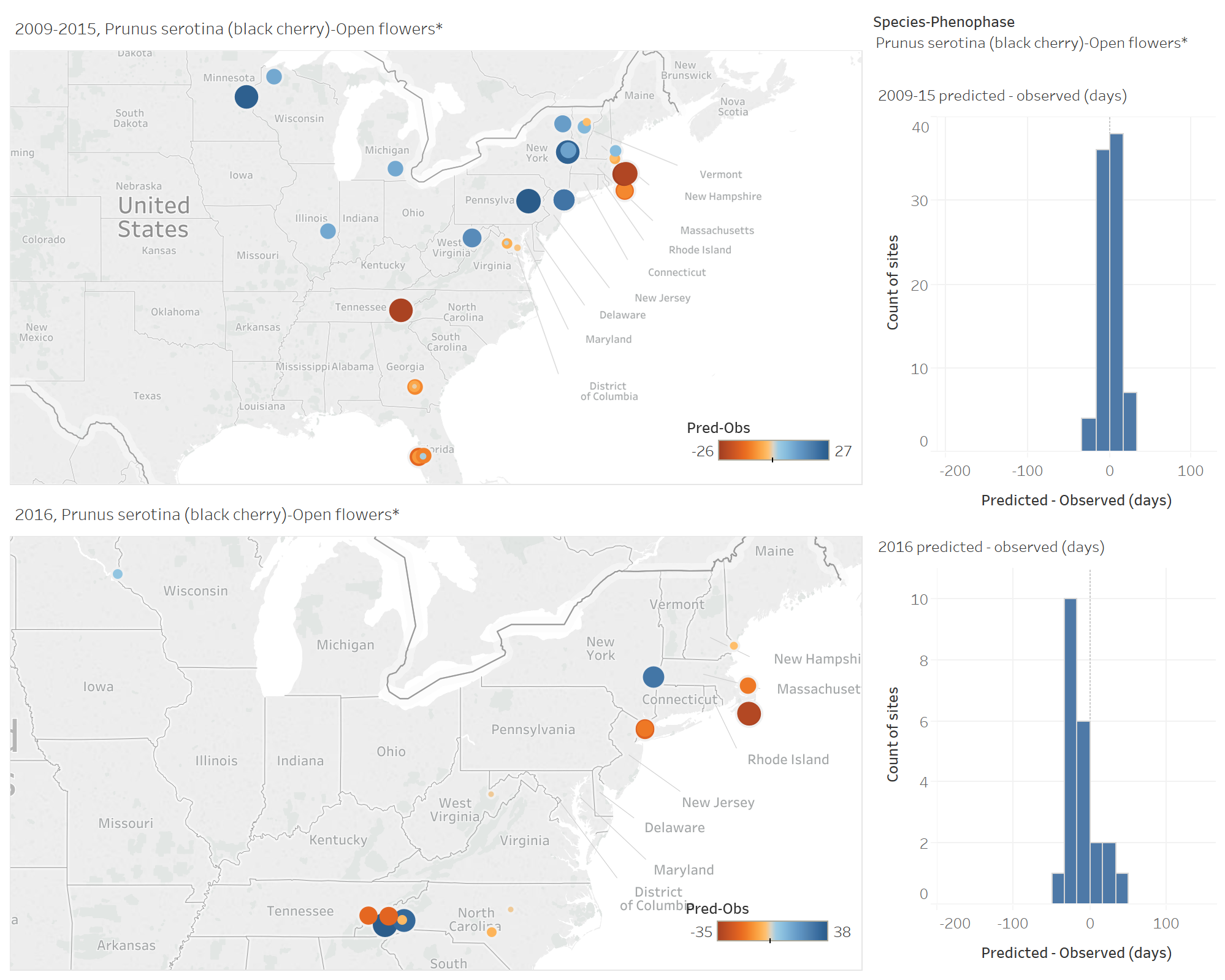

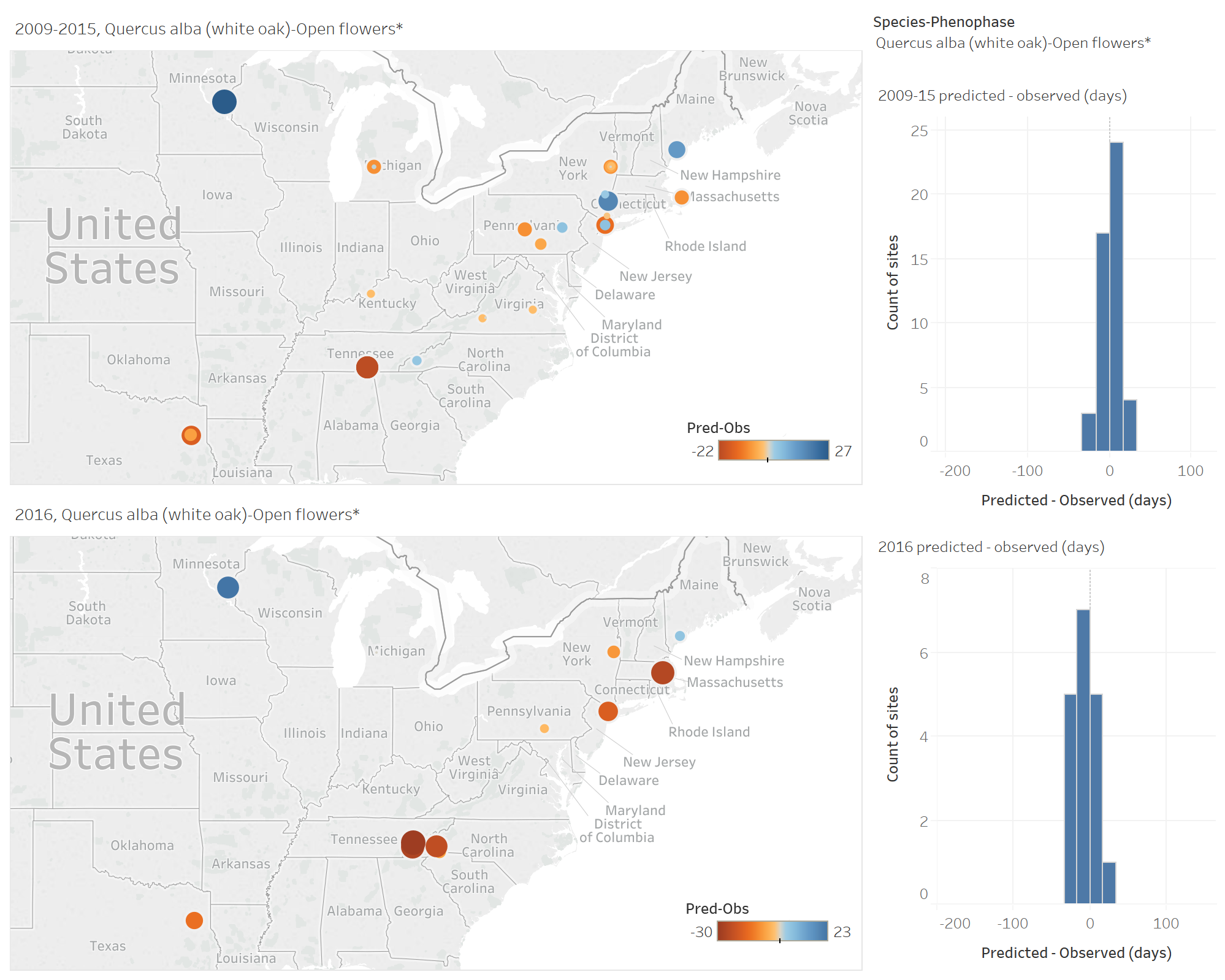

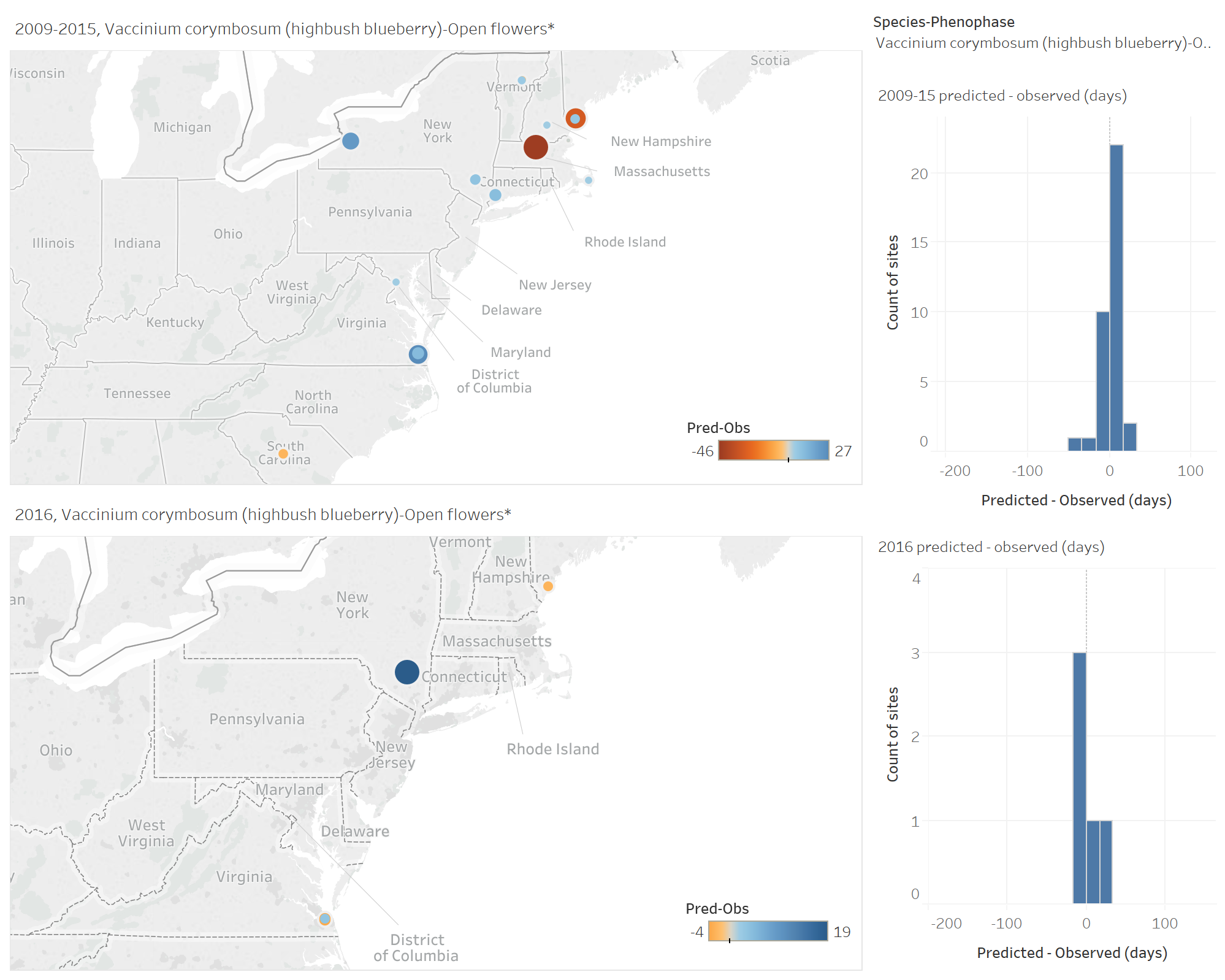

Supplement: S1 Appendix — Point size and color represent the difference, in days, between the predicted and the observed day of year for leaf-out. Locations where the model predicted leaves earlier than observer reports are shown in orange; locations where the model predicted leaves later than observer reports are shown in blue. Interactive maps available at https://tinyurl.com/usanpn-agdd-models. (DOCX) [file pone.0182919.s003.docx]
